# Obstructed infracardiac total anomalous pulmonary venous connection with patent ductus venosus: possibility of emergency palliation

**DOI:** 10.1186/s43044-025-00618-2

**Published:** 2025-02-21

**Authors:** Damandeep Singh, Niraj Nirmal Pandey, Joseph Thomas, Saurabh Kumar Gupta, Priya Jagia

**Affiliations:** 1 Department of Cardiovascular Radiology and Endovascular Interventions, All India Institute of Medical Sciences, New Delhi, 110029 India; 2 Department of Cardiology, All India Institute of Medical Sciences, New Delhi, 110029 India

**Keywords:** Obstructed infracardiac total anomalous pulmonary venous connection, Patent ductus venosus, Emergency palliation, Case report

## Abstract

**Background:**

Patients with obstructed infra-cardiac total anomalous pulmonary venous connection (TAPVC) require urgent intervention to relieve the obstruction, with or without restoration of anatomical continuity between the pulmonary veins and the left atrium. In cases of infra-cardiac TAPVC draining into the inferior vena cava (IVC) or hepatic vein, the obstructed channel can be accessed via the systemic venous approach for endovascular palliation. However, in cases of infra-cardiac TAPVC draining into the portal venous system, an endovascular approach to the obstructed channel is not possible via the transfemoral route and may require direct percutaneous puncture of the splenoportal axis.

**Case presentation:**

A 45-day-old boy presented with acute respiratory distress and cyanosis. CT angiography demonstrated infra-cardiac TAPVC with a focal critical stenosis in the descending channel, just proximal to its confluence with the portal vein. Incidentally, a vascular channel connecting the left branch of the main portal vein and the intra-hepatic IVC suggestive of a patent ductus venosus was noted. The patent ductus venosus would allow access to the site of obstruction (transfemoral venous approach → IVC → patent ductus venosus → left portal vein → main portal vein → obstructed descending common channel) to achieve emergency palliation by dilating the obstructed segment and subsequently, stenting the ductus venosus to circumvent the distal obstruction at the portal venous sinusoids.

**Conclusion:**

The present case highlights the role of CT angiography in delineating cardiovascular anatomy and demonstrating alternate vascular pathways that may be utilized for performing palliative endovascular procedures.

## Background

Total anomalous pulmonary venous connection (TAPVC) is characterized by anomalous drainage of all the pulmonary veins into the systemic or portal venous circulation, instead of the left atrium (LA). It represents approximately 1–5% of all congenital heart diseases. Infra-cardiac type of TAPVC comprises about 13–26% of all TAPVC cases and shows anomalous communication with either the systemic venous system (in about 10–20% cases) comprising hepatic veins, inferior vena cava (IVC), or the portal venous system (in about 80–90% cases). It is most frequently associated with obstruction due to inherent anatomical and physiological causes. Obstruction of the draining common channel leads to pulmonary venous hypertension and rapid deterioration, which may be associated with significant mortality. Patients with obstructed TAPVC require urgent intervention to relieve the obstruction with or without restoration of anatomical continuity between the pulmonary veins and the LA followed by definitive surgical repair. We describe a possible emergency palliation strategy in obstructed infracardiac TAPVC with a patent ductus venosus.

## Case presentation

A 45-day-old boy presented to the emergency room with acute respiratory distress and cyanosis. The child weighed 4 kg and had a history of poor feeding tolerance. On examination, the child had tachypnoea, chest retractions, and decreased blood oxygen saturation. Transthoracic echocardiography suggested a possibility of TAPVC after which the patient underwent ECG-gated CT angiography for further anatomical evaluation.

**Fig. 1:**
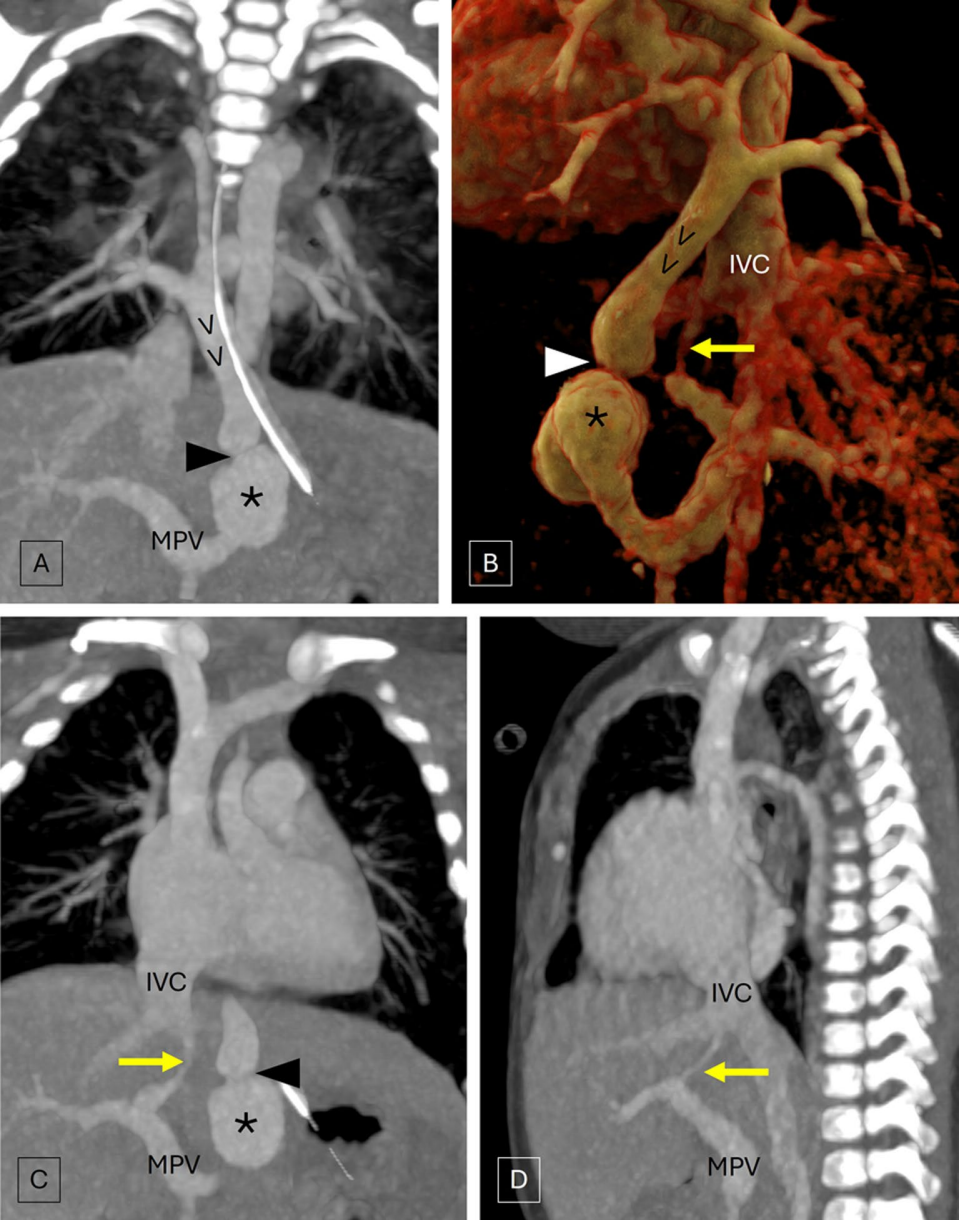
Coronal (**A**) and volume rendered images (**B**) of CT angiography depict the union of all four pulmonary veins forming a vertical vein (VV) that descends along the midline and drains into the main portal vein (MPV). Note is made of focal critical stenosis (arrowhead) in the descending channel, just proximal to its confluence with the MPV, with post-stenotic dilatation (*). The coronal (**C**) and sagittal (**D**) images demonstrate the patent ductus venosus (yellow arrow) connecting the left branch of the main portal vein and the intra-hepatic inferior vena cava (IVC)

CT angiography demonstrated the union of all four pulmonary veins forming a vertical vein that descended along the midline in the posterior mediastinum across the oesophageal hiatus and drained into the main portal vein suggestive of infra-cardiac TAPVC. There was focal critical stenosis in the descending channel, just proximal to its confluence with the portal vein, with post-stenotic dilatation. Incidentally, a vascular channel connecting the left branch of the main portal vein and the intra-hepatic IVC suggestive of a patent ductus venosus was noted (Fig. 1). Note was also made of marked smooth interlobular septal thickening in lungs with mosaic attenuation indicating pulmonary venous hypertension.

Subsequently, an emergent surgical correction was planned; however, the child was hemodynamically unstable and succumbed before he could be taken for surgery. Upon review of imaging, we hypothesized the possibility for endovascular palliation using the patent ductus venosus to access and relieve the obstruction in the vertical vein in patients which similar morphology who are hemodynamically unstable and/or cannot undergo an emergency surgical correction. This endovascular palliation may improve the functional status of patient and act as bridge to subsequent definite surgical correction.

## Discussion

In cases of supra-cardiac or cardiac TAPVC or cases of infra-cardiac TAPVC draining into the IVC or hepatic vein, the obstructed channel can be accessed via the systemic venous approach for endovascular palliation [1, 2]. However in cases of infra-cardiac TAPVC draining into the portal venous system, an endovascular approach to the obstructed channel is not possible via the transfemoral route and may require direct percutaneous puncture of the splenoportal axis.

**Fig. 2:**
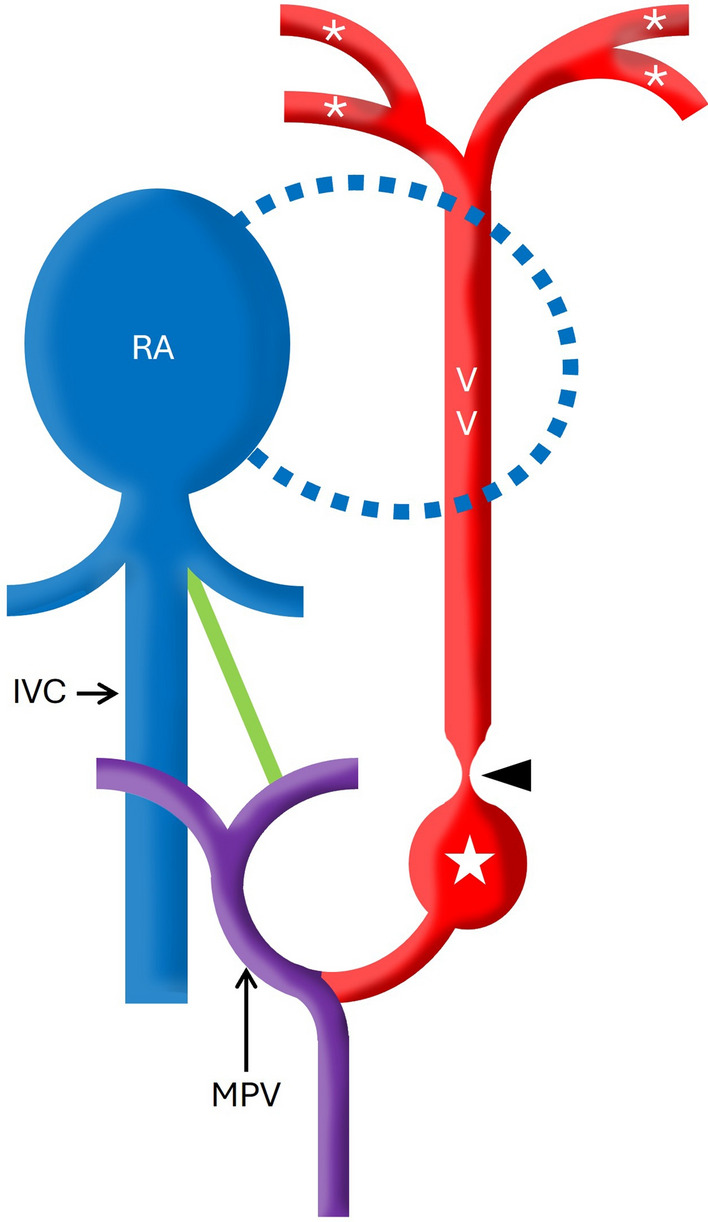
Schematic diagram shows the patent ductus venosus (in green) which allows access to the site of obstruction (arrowhead) via transfemoral venous approach which may be utilized to achieve emergency palliation. (star indicates the site of post stenotic dilatation; RA: right atrium; MPV: main portal vein; IVC: inferior vena cava; * indicates the pulmonary veins)

In the current case, the patent ductus venosus may allow access to the site of obstruction (transfemoral venous approach → IVC → patent ductus venosus → left portal vein → main portal vein → obstructed descending common channel) to achieve emergency palliation by dilating the obstructed segment and subsequently, stenting the ductus venosus to circumvent the distal obstruction at the portal venous sinusoids (Fig. 2) [3].

## Conclusion

In cases of obstructed infracardiac TAPVC, the definite management is surgical correction. However, the present case highlights the alternate vascular pathways that may be utilized for performing palliative endovascular procedures which can act as a bridge to subsequent definite surgical correction as well as the role of CT angiography in delineating the cardiovascular anatomy and demonstrating these alternate vascular pathways.

## Data Availability

Data availability is not applicable to this article as no new data were created or analysed in this study.

## Abbreviations

CT: Computed tomography
ECG-gated: Electrocardiographically gated
IVC: Inferior vena cava
LA: Left atrium
TAPVC: Total anomalous pulmonary venous connection
